# WHO malaria nucleic acid amplification test external quality assessment scheme: results of distribution programmes one to three

**DOI:** 10.1186/s12936-020-03200-0

**Published:** 2020-03-30

**Authors:** Jane A. Cunningham, Rebecca M. Thomson, Sean C. Murphy, Maria de la Paz Ade, Xavier C. Ding, Sandra Incardona, Eric Legrand, Naomi W. Lucchi, Didier Menard, Samuel L. Nsobya, Agatha C. Saez, Peter L. Chiodini, Jaya Shrivastava

**Affiliations:** 1 World Health Organization, Geneva, Switzerland; 2 Independent Consultant, London, UK; 3 Seattle Malaria Clinical Trials Center, Fred Hutchinson Cancer Research Center, Seattle, USA; 4 Department of Communicable Diseases and Health Analysis, Pan American Health Organization/World Health Organization, Washington, DC USA; 5 Foundation for Innovative New Diagnostics, Geneva, Switzerland; 6 Biology of Host-Parasite Interactions Unit, Institut Pasteur, INSERM U1201/CNRS ERL9195, Paris, France; 7 Malaria Branch, Division of Parasitic Diseases and MalariaCenter for Global Health, Centers for Disease Control and Prevention, Atlanta, USA; 8 Department of Pathology, School of Biomedical Science, Makerere University, Kampala, Uganda; 9 UK NEQAS Parasitology, Public Health England, London, UK; 10 The Hospital for Tropical Diseases, London, UK

**Keywords:** External quality assessment, Malaria, Molecular, Proficiency testing

## Abstract

**Background:**

The World Health Organization (WHO) recommends parasite-based diagnosis of malaria. In recent years, there has been surge in the use of various kinds of nucleic-acid amplification based tests (NAATs) for detection and identification of *Plasmodium* spp. to support clinical care in high-resource settings and clinical and epidemiological research worldwide. However, these tests are not without challenges, including lack (or limited use) of standards and lack of reproducibility, due in part to variation in protocols amongst laboratories. Therefore, there is a need for rigorous quality control, including a robust external quality assessment (EQA) scheme targeted towards malaria NAATs. To this effect, the WHO Global Malaria Programme worked with the UK National External Quality Assessment Scheme (UK NEQAS) Parasitology and with technical experts to launch a global NAAT EQA scheme in January 2017.

**Methods:**

Panels of NAAT EQA specimens containing five major species of human-infecting *Plasmodium* at various parasite concentrations and negative samples were created in lyophilized blood (LB) and dried blood spot (DBS) formats. Two distributions per year were sent, containing five LB and five DBS specimens. Samples were tested and validated by six expert referee laboratories prior to distribution. Between 37 and 45 laboratories participated in each distribution and submitted results using the online submission portal of UK NEQAS. Participants were scored based on their laboratory’s stated capacity to identify *Plasmodium* species, and individual laboratory reports were sent which included performance comparison with anonymized peers.

**Results:**

Analysis of the first three distributions revealed that the factors that most significantly affected performance were sample format (DBS vs LB), species and parasite density, while laboratory location and the reported methodology used (type of nucleic acid extraction, amplification, or DNA vs RNA target) did not significantly affect performance. Referee laboratories performed better than non-referee laboratories.

**Conclusions:**

Globally, malaria NAAT assays now inform a range of clinical, epidemiological and research investigations. EQA schemes offer a way for laboratories to assess and improve their performance, which is critical to safeguarding the reliability of data and diagnoses especially in situations where various NAAT methodologies and protocols are in use.

## Background

Malaria remains a global health challenge and despite recent advances still accounts for more than 400,000 deaths annually, with 90% of deaths occurring in Africa [1]. A key aspect of control and elimination of this disease is accurate diagnosis and since 2010 the World Health Organization (WHO) has recommended parasite-based diagnosis of malaria in place of syndromic treatment. Microscopy and rapid diagnostic tests (RDTs) are the main tools used for malaria case management and routine surveillance in endemic countries [2].

Over the last 30 years, important advances in molecular techniques have led nucleic acid amplification-based tests (NAATs) to be increasingly used for identification of *Plasmodium* infection. Abundant nucleic acid targets were first recognized in the 1980s by Waters and McCutchan [3]. Polymerase chain reaction (PCR) was first applied to *Plasmodium* by Snounou and colleagues in 1993 [4], which more easily enabled detection of *Plasmodium* nucleic acids in a blood sample. Since the advent of conventional PCR, numerous additional modifications have been developed, including nested PCR, real-time PCR, multiplex PCR, reverse transcription PCR, and loop-mediated isothermal amplifications (LAMP) [5–12].

These more sophisticated NAATs allow for species identification with use of species-specific primers or melt curve analysis, detection of mixed infections, and some NAATs can be used to estimate parasite density. NAATs have excellent sensitivity, down to 1–20 parasites/mL of whole blood in the most sensitive assays, depending on the volume of blood tested [12, 13], and permit earlier detection of infections compared to other diagnostic methods [9]. As NAATs can also be used to test archived material, samples can be conveniently analysed retrospectively.

NAAT have been increasingly used in epidemiological studies as well as the reference standard for malaria infection in evaluations of new diagnostic tests, in clinical trials assessing the efficacy of anti-malarial medicines and vaccines, and in clinical case management in high-income countries [14–16]. However, outside of these mostly resource-rich settings, the cost, specialist training and equipment requirements have largely restricted NAAT use to epidemiological and clinical trial research. In low-transmission endemic settings the greatest proportion of *Plasmodium falciparum* and *Plasmodium vivax* infections are at low density, below the limit of detection of microscopy and RDTs [17, 18], and such infections contribute to the transmission of malaria [19, 20]. In those areas, NAATs that enable the identification and treatment of such infections may accelerate elimination efforts such as screen and treat programmes and targeted malaria elimination strategies [21].

Many different NAAT methods are reported and are in use; however, there is a lack of or limited use of common standards used in different laboratories and a lack of formal external quality assessment (EQA) programmes for these methodologies [14].

Rigorous quality control is critical to ensuring that reliable and comparable results are generated by NAATs. Quality assessment activities, such as proficiency testing, can promote improvement in laboratory performance and alignment of methodology [22–25]. Amongst centres performing controlled human malaria infection (CHMI) studies, a previously reported EQA study showed high rates of agreement between centres [16]. However, beyond these highly regulated clinical trial sites, there is evidence of a lack of alignment between molecular diagnostic laboratories [26, 27], and there has not been wide uptake of international standards, such as the WHO DNA standard for *P. falciparum* [26, 28]. Malaria NAAT EQA or ‘proficiency testing’ schemes are a rarity, even in high-income countries [16]. EQA programmes for other malaria diagnostic tools, such as microscopy, have resulted in improved performance of these tests [29], while independent performance evaluation schemes of RDTs have resulted in shifts in the market towards better quality products [30].

Due to the heterogeneity of NAAT methodologies in use and reliance of policy making on research using NAATs as a reference standard, the WHO Global Malaria Programme commissioned the development of a repository of EQA materials for malaria NAATs. Together with the UK National External Quality Assessment Service (UK NEQAS) as the service provider, a NAAT EQA scheme informed by an expert consultation was started in January 2017 [31]. Participation was free of charge, and results were provided confidentially to the participating laboratories.

The scheme enables individual laboratories to assess their performance over time and, where necessary, troubleshoot and make improvements in their methodology. Across all participating laboratories, where factors associated with superior performance are evident, they are likely to promote harmonization of malaria NAATs among laboratories performing these techniques. Results from the first three distributions of this EQA scheme are presented in this paper, however the scheme is ongoing. The challenges encountered are also discussed, along with how the results of the scheme have been used by participating laboratories to improve their performance.

## Methods

### Enrolment of laboratories

Between December 2014 and January 2015, a survey regarding malaria NAAT activities was circulated widely through public health and research laboratory networks to reference and research laboratories in all WHO regions. Contact details were identified through a range of laboratory networks supporting clinical trials of medicines and vaccines, malaria elimination research and surveillance. Subsequently, 55 survey respondents who reported that they perform NAAT for *Plasmodium* detection were invited to participate in the EQA scheme, consisting of two distributions per year, on condition of signing a letter of agreement with WHO. Following presentations at international conferences and development of a WHO webpage featuring documentation describing the scheme, other laboratories joined in distributions 2 and 3 and more have joined in subsequent distributions.

### EQA source materials

Eighty-six per cent of *P. falciparum* and all *Plasmodium knowlesi* samples were sourced from in vitro cultures of laboratory strains prepared in the London School of Hygiene and Tropical Medicine (LSTMH). The other 14% of *P. falciparum* samples, as well as samples for other species, were prepared from leftover clinical specimens from patients attending the Hospital for Tropical Diseases in London and from residual blood samples referred from other hospitals across the UK. Under the terms of the UK Human Tissue Act, 2004, Schedule 4, Section 45, supplementary part 2, ethical approval to use such samples for EQA purposes is not required [32].

### Preparation of EQA samples

Methodology is described in detail elsewhere [33]. Briefly, leftover clinical samples consisting of EDTA-anti-coagulated peripheral blood were diluted in whole blood previously confirmed not to contain any *Plasmodium* parasites within 48 h of receipt from the diagnostic laboratory. Pre-dilution parasite densities were determined by expert microscopists counting the number of parasitized cells in a sample size of 10,000 red blood cells on thin blood films to obtain a percentage parasitaemia and converted to parasite density (number of parasites per µL) using the red cell count. For samples included in the first distribution, a red cell count of 5 × 10^12^ per litre was assumed, while red cell counts in subsequent distributions were determined in the initial, pre-dilution samples using a C-Chip DHC-N01 Disposable haemocytometer (NanoEnTek Inc. via MT Promedt Consulting GmbH, Germany). For cultured parasites, a thin blood film was made from the undiluted culture and a haemocytometer was used from the outset to obtain a red cell count in all cases, in order to obtain the pre-dilution parasite density in parasites per µL of synchronized ring-stage parasites.

For all samples, clinical and cultured, dilution to the desired parasite density was performed using parasite-negative whole blood supplied by UK National Blood and Transplant. To confirm the absence of *Plasmodium* in the supplied blood used for EQA sample preparation, only blood from seronegative donors was accepted, and blood used for negative samples underwent further PCR confirmation. All positive and negative samples were confirmed by PCR at multiple reference laboratories, again after sample production [5, 9, 34, 35]. Criteria for being a reference laboratory are outlined in the operational manual [33].

The range of parasite densities targeted was: 2 x 10^3^ parasites per microlitre of blood (p/µL), 2 x 10^2^ p/µL, 2 x 10^1^ p/µL, 2 p/µL, 0.2 p/µL, and 0.05 p/µL. Aliquots of the dilution series for each sample were then used to prepare dried blood spots (DBS) and/or lyophilized specimens.

To prepare DBS samples, 50 µL aliquots were deposited onto Protein Saver 903 cards (GE Healthcare Life Sciences) and air dried for 3–4 h in a tissue culture hood before being packaged in gas-impermeable bags with desiccant [33].

For lyophilization, each whole blood sample was aliquoted in 500 µL aliquots and lyophilized using a batch method and tray dryer, in a CHRIST freeze-dryer (EPSILON 2-12DS). Pre-freezing was carried out at − 40 °C for 3 h. Preparation for sublimation was carried out at − 30 °C for 30 min. Sublimation was done under the following conditions:

− 20 °C for 30 min at 0.040 mBar pressure
− 10 °C for 30 min at 0.040 mBar pressure
- 10 °C for 30 min at 0.030 mBar pressure
- 20 °C for 2 h at 0.025 mBar pressure

Secondary drying was carried out for 15 min at 20 °C and 0.025 mBar pressure. Vials were stoppered in a vacuum and crimp capped prior to storage and distribution. All samples are kept at − 80 °C for long-term storage.

### Panel composition

Each panel consisted of five DBS and five lyophilized blood (LB) samples and included both positive and negative samples. *Plasmodium falciparum* and *P. vivax* samples were sent in all three distributions, while *P. knowlesi* was included in distributions 1 and 2, and *Plasmodium malariae* in distribution 3 only (Table 1). Samples of these four species had parasite densities ranging from 0.018 to 800p/μL. There were no *Plasmodium ovale* samples included in distributions 1 to 3 due to a temporary supply shortage, but they have been included in subsequent distributions. Negative samples were included in all distributions in both DBS and LB formats.

**Table 1:** Characteristics of external quality assessment panels shipped to participants by distribution

Distribution	Lyophilized blood		Dried blood spots	
	Number of samples	Parasite density (parasites/µL)	Number of samples	Parasite density (parasites/µL)
Distribution 1—January 2017				
Negative	2	–	2	–
*P. falciparum*	0	–	3	0.05; 0.2; 2.0
*P. vivax*	2	0.018; 0.18	0	–
*P. knowlesi*	1	10	0	–
*P. malariae*	0	–	0	–
Distribution 2—July 2017				
Negative	1	–	2	–
*P. falciparum*	2	0.1; 1.0	0	–
*P. vivax*	1	0.018	0	–
*P. knowlesi*	1	1.0	3	2.0; 2.0; 20
*P. malariae*	0	–	0	–
Distribution 3—January 2018				
Negative	1	–	1	–
*P. falciparum*	2	20; 200	0	–
*P. vivax*	1	46.5	1	400
*P. knowlesi*	0	–	0	–
*P. malariae*	1	125	3	125; 400; 800

### Distribution

Panels of samples were shipped to participating laboratories twice a year. Distribution 1 panel was sent in January 2017, distribution 2 in July 2017 and distribution 3 in January 2018. Samples were sent via courier at ambient temperature directly to the majority of laboratories. In some cases they were sent via air freight to the nearest airport for collection.

### Reporting

After shipment of samples, participants were given 8 weeks to submit results via an online portal hosted by UK NEQAS. Participants were asked to use their usual methods for molecular analysis and to report extraction and amplification methods used and the result obtained for each sample. The options on the online portal for the individual samples included identification of each of the five species of human-infecting Plasmodia, the *Plasmodium* genus, a negative result, or an indeterminate result.

During the three distributions, seven laboratories failed to submit their results to the online portal by the submission deadline but provided results to the coordinators by email shortly thereafter. While these laboratories were not given an official report since they did not meet the deadline, results from these laboratories are included in this paper.

### Referee laboratories

Six referee laboratories were selected based on their technical qualifications, publication record using a range of malaria NAAT methods, available resources to serve as a referee laboratory, and geographic location [33].

### Analysis

Laboratories were asked to submit a ‘profile’ of their ability to identify positive samples to the genus level and to the species level for each of the five human-infecting *Plasmodium* species. Interpretation of the results was done by taking into account each site’s reported capacity to detect each species in order to ensure that laboratories would not be penalized for identifying false-negatives among samples that they do not have the capacity to detect routinely. In this paper, overall results are presented from all laboratories combined and performance is based on correct or incorrect results according to whether the result submitted matched the real sample, then adjusted according to the laboratory’s self-reported capacity to identify each species.

A full reporting scheme is available in the online operational manual [33]. Participants were encouraged to contest their scores, especially if they felt their profiling might have been wrong, but no complaints were received.

Data analysis was performed using Stata 15 (Stata Corp, College Station, USA). Factors which were found to have p < 0.1 in the univariate analysis were included in the multivariate model. For determining performance at high and low parasite density, a threshold of 2 p/µL was selected [36].

### Survey

In September 2018, after participants had experience with four distributions, laboratories were requested to complete an online survey with questions about problems they encountered during their participation in the scheme and if they had used their EQA results to amend the methodology or protocols in place at their site.

## Results

Overall, 57 laboratories had joined the WHO NAAT EQA scheme by distribution 3. Of these, 54 laboratories from 33 countries participated in distributions 1–3. Not all laboratories participated in all distributions. Participating laboratories included 11 (20%) from two countries in North America, 10 (19%) from nine countries in Africa, 12 (22%) from seven countries in Asia, 12 (22%) from eight countries in South/Central America, eight (15%) from six countries in Europe, and one (2%) in Australia.

Panels were shipped to 55 laboratories in distribution 1, 53 in distribution 2, and 56 in distribution 3. Forty-one laboratories submitted results in distribution 1, 37 in distribution 2, and 45 in distribution 3 (Table 2). More LB samples were analysed than DBS samples in all three distributions. Overall 359, 324 and 394 results were submitted from laboratories in distributions 1, 2, and 3, respectively.

**Table 2:** Characteristics of external quality assessment results submitted by each distribution

	Distribution 1	Distribution 2	Distribution 3
Panels shipped	55	53	56
Participants submitting results^a^	41 (75%)	37 (70%)	45 (80%)
Number of laboratories which processed			
Lyophilized blood	40 (98%)	35 (95%)	43 (96%)
Dried blood spots	32 (78%)	30 (81%)	36 (80%)
Number of samples submitted:			
Lyophilized blood	200	174	214
Dried blood spots	159	150	180
Total	359	324	394

^a^Results include samples from laboratories that submitted results after the EQA submission deadline

### Laboratory profiles

Of the 57 laboratories, 51 and 49 reported that they could analyse LB and DBS samples, respectively. All laboratories reported that they could identify *P. falciparum* to the species level. Seven (13%) laboratories reported that *P. falciparum* was the only species they could identify to species level, while nine (16%) could identify *P. falciparum* and *P. vivax* only to species level. Twenty-one (38%) laboratories reported that they could identify all species except *P. knowlesi*, one laboratory (2%) reported ability to identify all species except *P. ovale*, and 18 (32%) reported that they could identify all five species. The majority of laboratories reported that they could detect *Plasmodium* to genus level among species that they could not identify to species level, while some laboratories use species-specific primers, and are therefore unable to detect *Plasmodium* genus among samples of species that they do not aim to detect. For example, among the 31 laboratories that do not identify *P. knowlesi* to species level, 19 reported that they could identify *Plasmodium* among *P. knowlesi* samples.

### Methodology used by laboratories

**Fig. 1:**
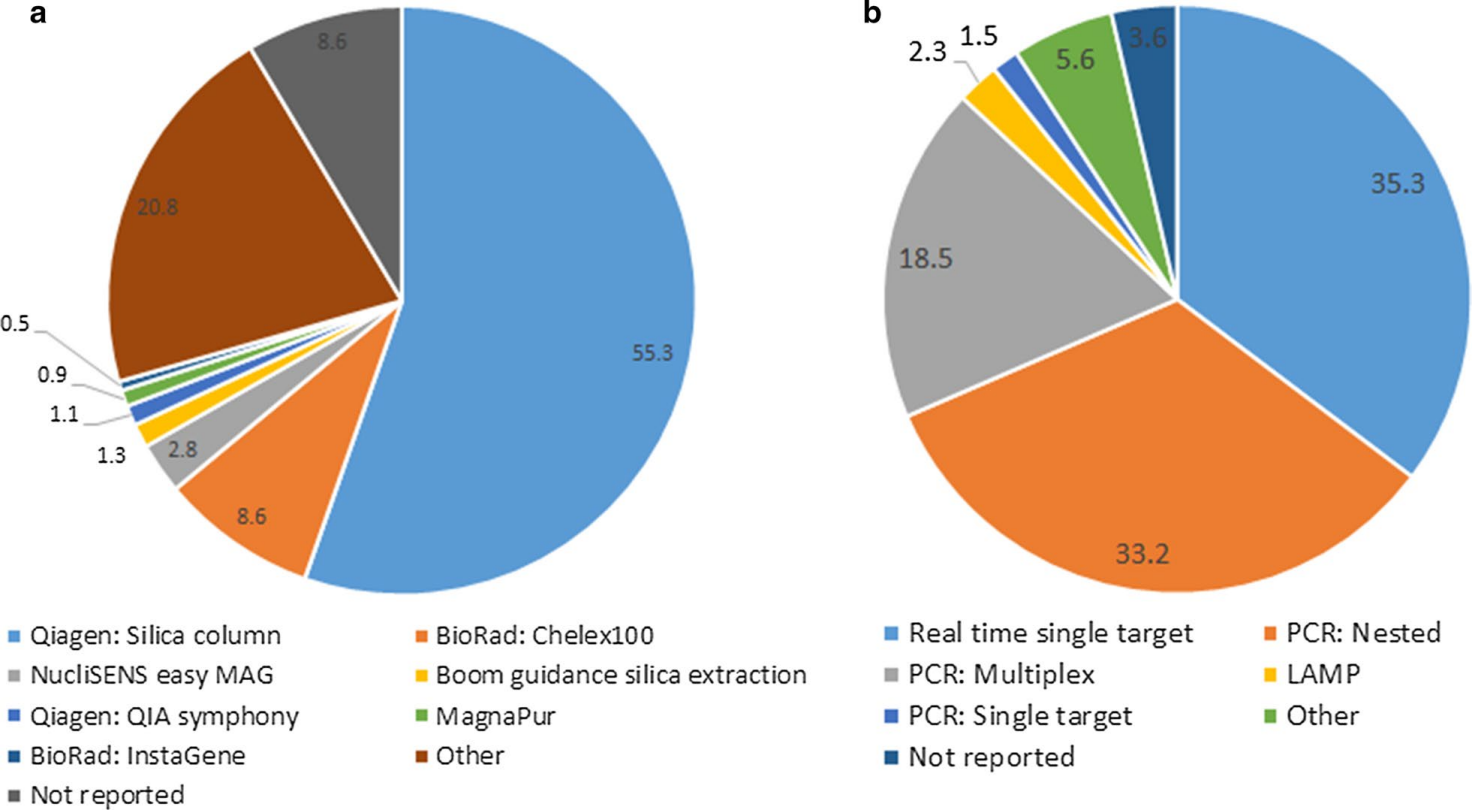
Characteristics of extraction (**a**) and amplification (**b**) methods used by participating laboratories across all three distributions. Percentages of laboratories for each methodology are shown. Methods of extraction and amplification included in other category were not described by participants

For nucleic acid extraction, use of a silica column was the most popular method, used in 55.3% of samples reported (Fig. 1). Chelex was used in 8.6% of samples, while 20.8% of samples were analysed using another method.

The most commonly used techniques for nucleic acid amplification were real-time single target PCR and nested PCR, used in 35.3 and 33.2% of samples analysed, respectively (Fig. 1). Multiplex PCR accounted for 18.5% of samples, while 2.3% of samples were analysed using LAMP and other techniques accounted for the remaining 10.7% of samples analysed. There was no significant difference in amplification technique used by distribution.

### Performance

**Fig. 2:**
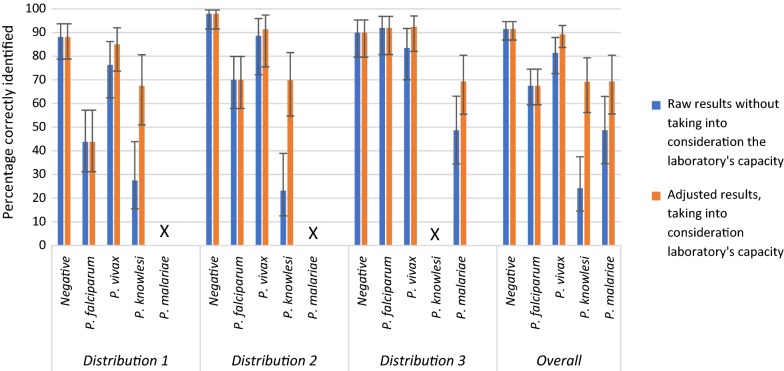
Accuracy of external quality assessment results by raw and laboratory capacity-adjusted results X: samples of this species were not included in this round

Based on three panels containing four of the five *Plasmodium* species infecting humans, across a parasite density range of 0.018-800 p/µL, the raw results and the results adjusted taking into consideration laboratory capacity are presented in Fig. 2.

When results were adjusted to take into account the laboratory’s reported capacity, the percentage of samples correctly identified as *P. falciparum* was unchanged as all laboratories reported that they had the ability to identify this species. The performance of correct *P. vivax* identification improved slightly to 89.2% upon adjustment, as only seven laboratories stated they did not have the techniques to identify *P. vivax*. The percentage of *P. knowlesi* samples correctly identified was 69.2%, 45.0% greater than using raw results, as 21 laboratories reported they could not identify *P. knowlesi*. Some 69.3% of *P. malariae* samples were correctly identified using adjusted results.

Based on adjusted results scores there was weak evidence that overall performance improved with each distribution, with 73.3, 80.5 and 83.0% of samples being correctly identified between distributions 1 and 3 (p = 0.09). The fewest false-positives in negative samples occurred in distribution 2, with 11.9, 2.1 and 10.1% of false-positives being reported in distributions 1, 2 and 3, respectively (p = 0.06). Detection of *P. falciparum* improved significantly from 43.8% in distribution 1 to 91.9% in distribution 3. Performance against *P. vivax* improved slightly, but not significantly, while neither *P. knowlesi* nor *P. malariae* samples were in all three distributions and therefore could not be evaluated over time.

**Fig. 3:**
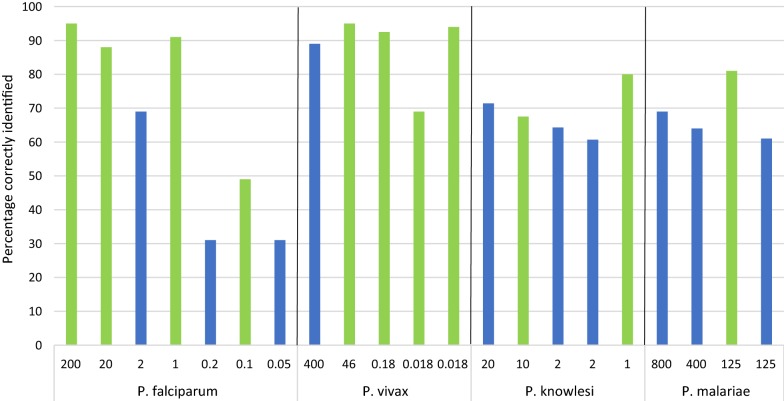
Accuracy of external quality assessment results on the basis of parasite species and density (parasites/µL). Results adjusted for laboratory capacity. Green bars denote lyophilized blood samples; blue bars denote dried blood spots

In Fig. 3, results were adjusted to match laboratory capacity for *Plasmodium* genus detection and species identification. As the concentration of *P. falciparum* decreased, generally the percentage of correctly identified samples also decreased, with 95.4% of the 200 parasite/µL samples being correctly identified and 31.3% of the sample at 0.05 parasites/µL being identified (p < 0.01). This trend was not seen for any of the other three species.

When applying a target NAAT detection threshold of 2 parasites/µL, adjusted performance was 79.1% against samples > 2 parasites/µL and 68.3% for samples ≤ 2 parasites/µL (p = 0.03) (Table 3). This was driven by the difference in performance against *P. falciparum* at different densities, with 91.7% of *P. falciparum* samples > 2 parasites/µL identified correctly and 54.8% of samples below this density (p < 0.01). There was no significant difference for *P. vivax* or *P. knowlesi*, and all samples of *P. malariae* were > 2 parasites/µL so a comparison could not be made. Performance against the higher concentration of DBS samples was 20.8 percentage points higher than the lower concentration; however, there was no significant difference against the LB samples.

**Table 3:** Accuracy of external quality assessment results above and below a density of 2 parasites/µL

	Percentage of samples correctly identified^a^		*P* value
	≤ 2 parasites/µL	> 2 parasites/µL	
Species			
*P. falciparum*	54.8	91.9	< 0.01
*P. vivax*	86.9	92.5	0.32
*P. knowlesi*	69.2	69.1	0.98
*P. malaria*^*b*^	NA	48.7	NA
Sample type			
Dried blood spots	50.1	70.9	0.02
Lyophilized blood	80.5	85.8	0.18
Overall	68.3	79.1	0.03

^a^Results adjusted for laboratory capacity

^b^All samples of *P. malariae* were > 2 parasites/µL

Participating laboratories detected lyophilized samples better than DBS samples, with 85.4 and 70.0% of samples, respectively, being identified correctly (p < 0.01) (Table 4). While performance was better than with DBS samples in all three distributions, the difference was only significant in distributions 1 and 3 (p < 0.01) and not in distribution 2 (p = 0.5).

**Table 4:** Result outcomes in relation to laboratory, test and sample characteristics

Characteristic	Number of samples analysed	% Correct based on results adjusted for laboratory capacity	*P*	Odds ratio unadjusted*	*P*	Odds ratio adjusted^a^	*P*
Region							
Africa	175	77.5 (69.4−84.0)	0.93	1	0.73		
Asia	250	78.8 (70.8−85.1)		1.1 (0.7−1.7)			
Europe	130	79.2 (68.6−87.0)		1.1 (0.6−1.9)			
North America	174	75.9 (64.9−84.2)		0.9 (0.6−1.5)			
South/Central America	319	79.3 (74.4−83.5)		1.1 (0.7−1.7)			
Oceania	29	82.8 (82.8−82.8)		1.4 (0.5−3.9)			
Reference laboratory							
Yes	114	83.3 (79.3−86.7)	0.06	1	0.07	1	0.02
No	963	78.5 (75.8−81.8)		0.7 (0.5−1.0)		0.7 (0.5−0.9)	
Type of sample							
Dried blood spots	489	70.0 (65.5–74.1)	<0.01	1	<0.01	1	<0.01
Lyophilized	588	85.4 (81.4−88.6)		2.5 (1.9−3.4)		2.3 (1.7−3.2)	
Species							
Negative	316	91.5 (86.8−95.6)	<0.01	1	<0.01	1	<0.01
*P. falciparum*	252	67.5 (59.5−74.5)		0.2 (0.2−0.4)		0.5 (0.2−1.4)	
*P. vivax*	194	89.2 (83.7−93.0)		0.8 (0.4−1.5)		2.6 (0.8−8.9)	
*P. knowlesi*	165	69.2 (56.9−79.3)		0.2 (0.2−0.4)		0.3 (0.1−0.8)	
*P. malariae*	150	69.3 (55.6−80.4)		0.2 (0.1−0.4)		0.2 (0.1−0.5)	
*Plasmodium* nucleic acid present							
Yes	761	73.8 (75.1−81.4)	<0.01	1	<0.01	–	
No	316	91.5 (86.8-94.6)		3.8 (2.5−5.8)			
Parasite density (parasites/µL)							
Negative	316	91.5 (86.8−94.6)	<0.01	1	0.01	1	0.03
>0– < 1	14	64.0 (56.0−71.4)		0.2 (0.1−0.3)		0.1 (0.04−0.4)	
≥1– < 10	162	74.1 (63.9−82.1)		0.3 (0.2−0.5)		0.7 (0.3−2.0)	
≥10– < 100	156	81.8 (74.4−87.4)		0.4 0.2−0.7)		0.7 (0.3−1.5)	
≥100	229	77.3 (84.2−93.3)		0.3 (0.2−0.5)		–	
Extraction method							
Qiagen: Silica column	596	79.9 (76.0−.83.3)	0.62	1	0.17		
BioRad: Chelex100	90	76.7 (64.0−85.9)		0.8 (0.5−1.4)			
NucliSENS easy MAG	30	90.0 (84.1−93.9)		2.3 (0.6−7.6)			
Boom guidance silica extraction	14	92.9 (92.9−92.9)		3.2 (0.4−25.3)			
Qiagen: QIA symphony	12	100		–			
MagnaPur	10	100		–			
BioRad: InstaGene	5	60.0 (60.0−60.0)		0.4 (0.1−2.3)			
Other	224	73.7 (65.7−80.3)		0.7 (0.5−1.0)			
Not reported	90	79.0 (59.4−90.5)		0.9 (0.5−1.6)			
Amplification method							
Real time single target	380	81.8 (76.3−86.3)	0.50	1	0.97		
Nested PCR	358	75.4 (68.6−81.2)		0.7 (0.5−1.0)			
Multiplex PCR	199	79.4 (71.2−85.7)		0.9 (0.6−1.3)			
LAMP	19	68.4 (41.2−87.0)		0.5 (0.2−1.3)			
Single target PCR	16	75.0 (70.3−79.2)		0.7 (0.2−2.1)			
Other	60	85.0 (67.8−93.9)		1.3 (0.6−2.7)			
Not reported	39	79.5 (57.6−91.7)		0.9 (0.4−2.0)			
Type of nucleic acid							
DNA	995	77.8 (74.3−80.9)	0.47	1	0.29		
RNA	45	84.4 (62.1−94.7)		1.6 (0.7−3.5)			

^a^Odds ratios (unadjusted and adjusted) show the ratio for each line compared to the first line in the section

Table 4 illustrates factors found significantly to affect adjusted performance, including parasite density, type of sample, species, and being a reference laboratory. The region in which the laboratory was located, type of nucleic acid amplified, amplification method, and extraction method did not significantly impact performance.

Overall adjusted performance varied between 20 and 100% across participating laboratories, with 46 (85%) laboratories correctly identifying > 70% of the samples that they tested. Twenty-six laboratories participated in all three distributions. Of these, seven laboratories (27%) scored 80% or higher in all three distributions, while 19 (73%) laboratories scored 60% or higher in all three distributions.

### Challenges for EQA participants and the provider

Most laboratories (85%, 46/54) surveyed provided information about challenges they faced during their participation in the scheme. The main challenge related to delays in obtaining samples, mainly due to customs requirements, often compounded by the airport collection point being far from the laboratory. Out of the laboratories that completed the survey, nine (20%) reported consistently having problems with shipment delays while five laboratories (11%) reported having problems in only some of the distributions. Problems with entering data into the submission portal were reported. Other challenges included not having any or sufficient quantities of reagents or laboratory supplies to analyse the samples before the deadline and not having personnel available for various reasons (e.g., staff in the field).

From an EQA provision perspective there were numerous challenges, mostly related to identifying the correct contact person for each laboratory, issues related to import permits, and identifying relevant courier partners for shipment to certain geographic regions. Language barriers and working across time zones added to the complexity. The reporting time allowed was extended in the first distribution in order to accommodate these difficulties.

### How laboratories have used their EQA results

Of 46 laboratories that provided information about their participation, 13 (31%) responded that they changed or amended their laboratory protocol(s) in response to their results from the EQA scheme. Of these laboratories, changing the protocol of the extraction and/or amplification processes were the main responses (four laboratories), while two other laboratories reported adding new PCR methods to their existing methods. Three laboratories reported that the scheme allowed them to identify additional species that they previously did not identify by testing and refining their protocols for these new species during the scheme. Two laboratories reported adding *P. knowlesi* identification and one other laboratory *P. knowlesi, P. malariae* and *P. ovale*.

## Discussion

The use of NAATs for malaria diagnosis in research and clinical care has increased dramatically over the last two decades. Clinical trials of new anti-malarial drugs or vaccines require precise, reliable methods for parasite detection, and low density of parasitaemia may be missed by routine diagnostic methods, such as microscopy and RDTs. NAAT can also be used for high throughput of samples which is not possible using other methods. The continual evolution of NAATs and the lack of standardized methods highlight the need for an EQA scheme to ensure safe laboratory performance and, over the longer term, potentially to facilitate assay harmonization. The scheme is also a mechanism to promote use of and reporting based on the available International DNA Standard and to advocate for the creation of more such standards (e.g., the need for an International Standard for RNA-based assays).

Upon recommendations of WHO and building on the experiences of UK NEQAS and others in the research community [16, 31, 37], this new international scheme is unique in its geographic coverage, panel sample format and composition and breadth of participation. The scheme thus allows laboratories to compare their performance with their global peers, across various specimen types, including all five malaria species and, over a suitable time period, to assess the effect of changes that might have been introduced as a direct effect of participation within this EQA scheme. Although data from more distributions will be needed to draw broader conclusions and assess the impact, this analysis already presents some interesting findings and challenges.

Firstly, false-positives against malaria-negative samples were less common than false-negatives against *Plasmodium*-containing samples. The false-positive rate was 8.5% compared to a 26.2% false-negative rate. The higher false-negative rate likely reflects the lower parasite densities of distributed *P. falciparum* samples since higher density samples > 2 parasites/µL were more reliably detected (e.g., 7.5% false-negative rate for such *P. vivax* samples and 8.1% false-negative rate for such *P. falciparum* samples). These data show that misalignment in laboratory results can be expected, especially as the parasite density goes down.

The scheme included DBS because this is the most common sample format used in research in endemic countries due to its low cost, ease of transport and storage, and stability [38]. Performance was significantly higher against 500 µL LB samples than 50 µL DBS, especially at concentrations below 2 p/µL. This is consistent with reported findings [39] and needs to be taken into consideration when comparing results of studies using different sample types. Inclusion of paired samples in future distributions will allow for a more direct comparison between performance of DBS and LB samples. Laboratories were not asked to provide references or specific protocol details, such as how much of the LB and/or DBS sample was tested in their protocol. Requesting this information could allow the effect of sample quantity used on performance to be assessed.

All of the factors that were found to significantly affect performance were due to the characteristics of the samples themselves (format, species, parasite density), and not the laboratory location (geography) or the reported methodology used (nucleic acid extraction, amplification, or DNA *vs* RNA target) with the exception that referee laboratories performed better than non-referee laboratories. This would suggest that referee laboratories could play an important role in shaping malaria molecular testing practice through publication of protocols and mentoring.

To overcome the challenges identified by participants, some revisions were implemented in the EQA scheme, including a requirement to supply copies of import permits prior to shipment, translation of key documents into host country language and, in some cases, centralized shipment to a WHO country office for subsequent onward shipment to participating laboratories. The turn-around time for reporting results was extended from 6–8 weeks. Participation was also restricted to those laboratories regularly conducting NAATs to avoid failure to submit results caused by lack of reagents, lack of personnel, or lack of ongoing studies to support testing. The online data entry interface was adapted based on feedback to improve user-friendliness. In response to challenges brought to the attention of the EQA provider, various changes were incorporated, including creating a separate inbox for query handling, sending reminder emails to participants for import permits and other relevant documentation, including a list of laboratory profiles toward the end of EQA reports for each distribution, to illustrate the rationale for scoring and rewording the information section of the participant portal better to explain the data entry procedure.

As a result of their involvement in the scheme, several laboratories amended their protocols or included new methods, with some laboratories including new species into the range tested. One important aim of the scheme was to enable laboratories to learn from their results and from each other on ways to improve performance.

Certain limitations of the study must be acknowledged. As not all laboratories participated in all distributions, and due to the different panel composition during each distribution, both in terms of species and parasite density, it is not possible to compare performance directly over time with only three distributions. As the EQA scheme progresses and more data are collected, it will be interesting to assess whether there are more significant changes in performance over time, best demonstrated by trends in individual laboratories. Samples were diluted and parasite densities determined by UK NEQAS. Calibration of EQA materials with the International DNA Standard could also be performed and is done in one of the referee laboratories. However, all International Standards are expressed in International Units, whereas clinical laboratories and WHO work in parasites/volume or copies/volume. The EQA programme chose a density threshold of 2 p/µL to determine ‘adequate’ performance, the sensitivity of a test required to detect asymptomatic infections and those in low-density populations [36]. The sensitivity required of a test can depend on the purpose of testing, with target sensitivity for drug and vaccine efficacy trials being greater than that required for case management, so perhaps one threshold for a given assay is not sufficient as an indicator of its utility. For instance, the limit of detection for assays intended to support CHMI studies is generally lower (0.01–0.05 parasites/µL). However, whether the scheme uses one or more thresholds, the critical point is that individual laboratories may use this scheme to obtain objective performance data, including the limit of detection for the test(s) they use and undertake quality improvement by investigating any failures. These will all help them select assays appropriate for the specific samples they are asked to examine.

This scheme intends to evolve in the constantly changing malaria landscape. For example, future distributions of the scheme will include *pfhrp2*- and *pfhrp3*-negative *P. falciparum* samples, allowing laboratories that analyse these genes to assess their accuracy at identifying samples with gene deletions. It is critical that surveys for *pfhrp2/3* gene deletions should yield accurate and reliable results, as over- or under-calling their presence will have a significant impact on policy decisions on the most appropriate malaria RDTs to be used in these areas.

While the scheme is currently free for all participating laboratories, this arrangement is unsustainable in the long term. In the near future, user fees will be introduced to cover costs, including the replenishment of EQA materials. Costs are expected to be between US$300–350 per laboratory per distribution, which is an extremely small fraction of the cost of clinical and epidemiological research budgets, and a small investment to safeguard against decision-making based on flawed data. Donors funding malaria research and laboratory capacity strengthening should require laboratories to participate in EQA schemes, as is a requirement for reference laboratories in countries with mature quality assurance systems. For laboratories that do not originate from low or middle income countries (LMICs) or participate in both the UK NEQAS and WHO schemes, participation in the UK NEQAS Malaria molecular scheme will be encouraged so that more laboratories from LMICs can participate in the WHO scheme. Thus, ongoing EQA for malaria NAATs can help to safeguard clinical trial, clinical care (in some high-resource settings), and research activities that rely on these increasingly important tests.

## Conclusions

William Osler is quoted as saying “As is our pathology, so is our practice” [40]. As the range and sophistication of diagnostic tests has increased, so has the relevance of his comment. Furthermore, at long last, reliable, quality-assured diagnostic tests for malaria are available for use in resource-poor settings. As technology advances yet further, that repertoire will increasingly include NAAT. But to deploy them without ensuring good performance on the ground would be a major error that could adversely influence decisions affecting both individual patients and whole populations.

## Data Availability

The datasets used and analysed during the current study are available from the corresponding author on reasonable request.

## Abbreviations

CHMI: Controlled human malaria infection
DBS: Dried blood spot
DNA: Deoxyribonucleic acid
EQA: External quality assessment
LAMP: Loop-mediated isothermal amplifications
LB: Lyophilized blood
LSHTM: London School of Hygiene and Tropical Medicine
NAAT: Nucleic acid amplification technique
PCR: Polymerase chain reaction
RDT: Rapid diagnostic test
RNA: Ribonucleic acid
WHO: World Health Organization
